# Epstein–Barr virus antibody level and gastric cancer risk in Korea: a nested case–control study

**DOI:** 10.1038/sj.bjc.6605146

**Published:** 2009-06-23

**Authors:** Y Kim, A Shin, J Gwack, K-P Ko, C-S Kim, S K Park, Y-C Hong, D Kang, K-Y Yoo

**Affiliations:** 1 Cancer Early Detection Branch, National Cancer Control Research Institute, National Cancer Center, 111 Jungbalsan-ro, Ilsandong-gu, Goyang-si, Gyeonggi-do 410-769, Korea; 2 Cancer Epidemiology Branch, Division of Cancer Epidemiology and Management, Research Institute, National Cancer Center, 111 Jungbalsan-ro, Ilsandong-gu, Goyang-si, Gyeonggi-do 410-769, Korea; 3 Department of Preventive Medicine, Seoul National University College of Medicine, 103 Daehangno, Chongno-gu, Seoul 110-799, Korea; 4 Division of Epidemiology and Health Index, Center for Genome Science, Korea Center for Disease Control and Prevention, 194 Tongil-ro, Eunpyeong-gu, Seoul 122-701, Korea; 5 Seoul National University Cancer Research Institute, 103 Daehangno, Chongno-gu, Seoul 110-799, Korea

**Keywords:** stomach neoplasms, Epstein–Barr virus, nested case–control study, Korea

## Abstract

**Background:**

Few cohort studies have investigated Epstein–Barr virus (EBV) infection before the occurrence of gastric cancer.

**Methods:**

Among 14 440 cohort participants, 100 incident gastric cancer cases were individually matched to two controls. Epstein–Barr virus antibodies IgG and IgA against viral capsid antigen (VCA), EBV nuclear antigen (EBNA) antibody IgG, and early antigen (EA) antibody IgG were measured using enzyme immunoassays (EIAs).

**Results:**

The highest titres of VCA IgG (odds ratio (OR): 1.37, 95% confidence interval (CI): 0.62–3.06) or EBNA IgG (OR: 0.87, 95% CI: 0.51–1.46) were not associated with gastric cancer risk.

**Conclusion:**

Higher levels of VCA IgG or EBNA IgG were not associated with increased risk of gastric adenocarcinoma in Koreans.

Epstein–Barr virus was classified as a group I carcinogen for Burkitt's lymphoma, nasopharyngeal cancer, Hodgkin's and non-Hodgkin's lymphoma (IARC, 1997), and the elevated levels of EBV capsid antigen (VCA) and EBV early antigen (EA) were detected before the development of Burkitt's lymphoma (Geser *et al*, 1982), nasopharyngeal cancer (Chien *et al*, 2001), Hodgkin's and non-Hodgkin's lymphoma (Lehtinen *et al*, 1993), and testicular cancer (Akre *et al*, 1999; Macsween and Crawford, 2003).

Epstein–Barr virus infection is believed to be related to 5–16% of gastric adenocarcinomas, with variations across populations (Burgess *et al*, 2002; Macsween and Crawford, 2003; Correa *et al*, 2004; Herrera-Goepfert *et al*, 2005). Most studies to date have been tumour tissue-based, providing information about the role of EBV in gastric carcinogenesis, but providing limited information on the association between EBV infection and gastric cancer, particularly adenocarcinoma – the most prominent histology (Tokunaga and Land, 1998; Chang and Kim, 2005; Akiba *et al*, 2008). The few epidemiological studies of EBV infection and gastric cancer, have mainly been limited by small size and non-prospective design (Levine *et al*, 1995; Shinkura *et al*, 2000). A recent case–control study with 185 gastric cancers did not take account of the possible contribution of *Helicobacter pylori* to the question (Koshiol *et al*, 2007).

We carried out a nested case–control study within the Korean Multi-Center Cohort (KMCC) study of the possible relationship between EBV-specific antibody titres and gastric cancer, adjusting for *H. pylori* infection status.

## Materials and methods

Individuals in this nested case–control study were participants in the KMCC (Yoo *et al*, 2002; Gwack *et al*, 2006). Men and women aged ⩾30 years were recruited from 1993 to 2002. Informed consent was obtained from all participants who were enrolled in this study, and the Institutional Review Board of Seoul National University Hospital approved the study protocol (Review No. C-0603-161-170). Blood samples were collected after an overnight fast, processed within 8 h, stored at −20°C for 2–3 days, and transferred to a central site where they were preserved at −80°C.

A total of 14 440 men and women aged ⩾30 years were recruited from 1993 to 2002. Incident cancer cases were identified by a computerised record linkage to the Korea Central Cancer Registry database and the National Health Insurance database using resident registration numbers. The last date of follow-up was 31 December 2002, and 100 incident gastric cancer cases were identified among the 14 440 cohort members by the end of the follow-up period. Among participants who were alive and had no period of follow-up loss at the date when each case was diagnosed, controls were selected and matched 1 : 2 by incidence density sampling for the year of enrolment, area of residence, age within 5 years, and gender. The follow-up period was defined as the date of recruitment to the end of follow-up or to death. For the case group, the end of the follow-up date was defined as the date of diagnosis from the Korea Central Cancer Registry database.

Four EBV-specific antibody titres were tested using commercially available enzyme-linked immunosorbent assays (ELISA), namely EBV viral capsid antigen (VCA) IgG (EBV VCA IgG; Zeus Scientific, Raritan, NJ, USA), EBV VCA IgA (EBV VCA IgA; IBL, Hamburg, Germany), EBV nuclear antigen (EBNA) IgG (EBNA IgG; Zeus Scientific), and EBV EA IgG (EBV EA IgG; IBL).

The laboratory was blinded to the case–control status. For quality control of EBV VCA IgG and EBNA IgG, one negative and one positive control was included in each plate of 96-wells. Sensitivity for EBV VCA IgG was reported to be 98.3% and specificity 98.1% compared with an immunofluorescence assay. The sensitivity for EBNA IgG was reported to be 94.4% (90.0–97.3%) and specificity 98.9% (93.8–100%) compared with another ELISA system currently in commercial distribution. Four kinds of control samples were used for quality control of EBV VCA IgA and EA IgG, according to the manufacturer's guidelines: one negative control, one cutoff control, one weakly positive control, and one positive control in each plate. The relative sensitivity and specificity for EBV VCA IgA and EA IgG was reported to be >95% compared with another ELISA method. These ELISA methods from various companies have been shown to correlate closely with immunofluorescence serology (Bruu *et al*, 2000).

Seropositivity for *H. pylori* antibody was identified by testing the expression of *H. pylori*-specific antibody IgG using immunoblot kits (Helico Blot 2.1; MP Biomedicals Asia Pacific, Singapore, Singapore).

Antibody titres were categorised into binary variables as negative or positive, including indeterminate as positive for statistical analysis. The titres of VCA IgG and EBNA IgG were re-categorised into three tertile groups based on titres of control groups.

Odds ratios (ORs) and 95% confidence intervals (CIs) were calculated using a multivariate conditional logistic regression model. The SAS software version 9.1 (SAS Institute, Cary, NC, USA) was used for all statistical analyses.

## Results

Table 1 shows the basic characteristics between cases and controls. Adenocarcinoma comprised 87% of the gastric cancer cases, 2% were other tumours, including single cases of malignant carcinoid tumour and squamous cell carcinoma *in situ*; 11% were unspecified.

Viral capsid antigen IgG, which develops early in EBV infection and persists throughout life, was positive in 94% of the cases and in 92% of the controls (*P*=0.4). Epstein–Barr virus nuclear antigen IgG, another antibody indicating latent infection, was positive in 79% of the cases and in 82% of the controls (*P*=0.4). Viral capsid antigen IgA, an indicator of recent infection, was positive in 2% of the cases and in 3% of the controls. Lytic enzyme EA IgG was positive in 12% of the cases and in 11% of the controls.

Crude and adjusted ORs for VCA IgG, VCA IgA, EBNA IgG, and EA IgG of indeterminate and positive categories compared with the negative category were not significantly associated with gastric cancer risk (Table 2). This remained the case when VCA IgG and EBNA IgG positive groups were re-categorised by the titre level of below and above median, higher titre levels. When the analysis was restricted to adenocarcinoma case pairs, the OR for the highest tertile of VCA IgG was 1.33 (95% CI: 0.59–3.00) and that of EBNA was 0.92 (95% CI 0.53–1.58) compared with the negative category (Table 3). For gastric adenocarcinoma pairs, including those who were diagnosed more than 2 years after enrolment, the ORs for the highest category of VCA IgG and EBNA IgG *vs* the negatives were 1.53 (95% CI: 0.62–3.75) and 1.23 (95% CI: 0.64–2.36), respectively.

## Discussion

In this study, more than 80% of the study participants tested positive for EBV latent infection antibodies (EBV VCA IgG and EBNA IgG), and no significant association between seropositivity for latent EBV infection and gastric cancer risk was observed. Even after restricting the analysis to adenocarcinoma pairs who were in follow-up more than 2 years after enrolment, there was no significant association between antibody levels and gastric cancer risk.

The highest category of VCA IgG and EBNA IgG titres had an increased risk of gastric adenocarcinoma in a nested case–control study of 54 cases and controls, but the difference was not significant (Levine *et al*, 1995). A recent Chinese nested case–control study suggested an opposite pattern, that a high VCA IgG level might be associated with a lower risk of cardiac gastric cancers (Koshiol *et al*, 2007). In our study, EBV latent infection antibodies were not associated with gastric cancer risk. These differences may be due to infection by different EBV strains, which are believed to influence EBV-infected gastric cancer worldwide in combination with different environmental factors (Herrera-Goepfert *et al*, 2005).

Another possible reason for these inconsistencies may be an interaction between *H. pylori* and EBV infection, which is poorly understood. In this study, we measured seropositivity of *H. pylori* antibody and found that the interactions between seropositivity for *H. pylori* and EBV VCA IgG and EBNA IgG were not statistically significant (*P*=0.87 and *P*=0.79, respectively; data not shown). However, an interaction between both infections might be plausible, as both *H. pylori* and EBV infection have a role in carcinogenesis in the pathway of inflammation, chronic infection, and autoimmune pathway (Ouburg *et al*, 2005; Gwack *et al*, 2006). Several candidate gene approach studies suggest that EBNA genes interact with inflammatory and tumour suppressor genes, which has implications in gastric carcinogenesis (Ouburg *et al*, 2005).

In the analysis of EBNA, EA, and VCA antibodies, immunofluorescence assay methods are more time consuming and difficult to interpret, but are more specific, have high resolution, and stand as the ‘gold standard’ technique. Enzyme immunoassay (EIA) is often used in clinical laboratories because of its reliability in high-throughput analysis (Gartner *et al*, 2003). The validity of the EIA kits is supported by reasonably high sensitivity (95–100%) and specificity (86–100%), compared with the indirect immunofluorescence assay as a gold standard (Bruu *et al*, 2000; Gartner *et al*, 2003). Large-scale population-based studies have recently used EIA (Tedeschi *et al*, 2007).

In our study, pathological specimens were not collected so that the association between EBV antibody titre and gastric cancer was not compared among EBV-related and non-EBV-related gastric cancer cases. We cannot exclude chance effects due to our small numbers, and further studies are required. Antibodies were checked only at the time of enrolment and not re-tested, which omitted detailed information about EBV infection status during follow-up.

Our study has several strengths; it was nested in a prospective cohort study, and sera were collected before the diagnosis of gastric cancer, which may minimise any possibility of misclassification for exposure. We restricted our analysis to gastric adenocarcinoma cases. If lymphoepithelioma-like cancer was included among the ‘unspecified’ pathological group, the OR may have tended towards null. To exclude potentially developing cases before or near the period of enrolment, we restricted our analysis to individuals who were diagnosed after 2 years of enrolment.

In summary, our results provide little support for the hypothesis that increased levels of EBV antibodies are related to gastric adenocarcinoma.

## Figures and Tables

**Table 1:** Baseline characteristics of the patients with gastric cancer and controls

	**No. (%)**		
**Variables**	**Cases (*n*=100)**	**Controls (*n*=200)**	***P*-value**
*Age at enrollment (years)*			0.49^a^
⩽59	31 (31.0)	49 (24.5)	
60–69	46 (46.0)	100 (50.0)	
⩾70	23 (23.0)	51 (25.5)	
Male (%)	67	67	—
			
*Cigarette smoking*			0.32^a^
Never	40 (40.0)	81 (40.5)	
Past	14 (14.0)	32 (16.0)	
Current	46 (46.0)	87 (43.5)	
			
*Alcohol consumption*			0.42^a^
Never	50 (50.0)	101 (50.5)	
Past	12 (12.0)	15 (7.5)	
Current	38 (38.0)	84 (42.0)	
			
*Education (years)*			0.65^b^
None	27 (27.0)	64 (32.0)	
1–12	71 (71.0)	133 (66.5)	
⩾13	2 (2.0)	3 (1.5)	
			
*H. pylori* seropositivity (%)	89 (89.0)	181 (90.5)	0.68^a^
			
*Follow-up duration (years)*			—
<2.0	37 (37.0)		
2.0–4.9	46 (46.0)		
⩾5.0	17 (17.0)		
			
*Tumor sites*			—
Cardia	7 (7.0)		
Non-cardia	75 (75.0)		
Unspecified	18 (18.0)		
			
*Pathologic type*			—
Adenocarcinoma	87 (87.0)		
Others	2 (2.0)		
Unspecified	11 (11.0)		

*H. pylori*=*Helicobacter pylori*.

a *χ*^2^-test.

b Fisher's exact test.

**Table 2:** Odds ratios of gastric cancer risk according to the Epstein–Barr virus (EBV) antibody titres

**Antibody**	**Category**	**Cases (*n*=100) no.**	**Controls (*n*=200) no. (%)**	**cOR**	**(95% CI)**	**aOR**	**(95% CI)^a^**
VCA IgG	Negative	3	11 (5.5)	1.00	—	1.00	—
	Indeterminate or positive	97	189 (94.5)	1.26	(0.60–2.62)	1.24	(0.59–2.62)
VCA IgA	Negative	98	194 (97.0)	1.00	—	1.00	—
	Indeterminate or positive	2	6 (3.0)	0.87	(0.35–2.15)	0.83	(0.33–2.09)
EBNA IgG	Negative	19	31 (15.5)	1.00	—	1.00	—
	Indeterminate or positive	81	169 (84.5)	0.88	(0.56–1.39)	0.85	(0.54–1.36)
EA IgG	Negative	88	178 (89.0)	1.00	—	1.00	—
	Indeterminate or positive	12	22 (11.0)	1.04	(0.65–1.65)	1.07	(0.67–1.72)
							
VCA IgG	Negative	3	11 (5.5)	1.00	—	1.00	—
	Seropositive, below median	51	94 (47.0)	1.28	(0.61–2.71)	1.27	(0.60–2.72)
	Seropositive, above median	46	95 (47.5)	1.23	(0.58–2.61)	1.21	(0.56–2.60)
EBNA IgG	Negative	19	31 (15.5)	1.00	—	1.00	—
	Seropositive, below median	36	84 (42.0)	0.86	(0.53–1.38)	0.81	(0.50–1.32)
	Seropositive, above median	45	85 (42.5)	0.92	(0.56–1.50)	0.90	(0.55–1.49)
							
VCA IgG	Negative	3	11 (5.5)	1.00	—	1.00	—
	1T (lowest)	36	65 (32.5)	1.28	(0.60–2.70)	1.27	(0.59–2.72)
	2T	24	64 (32.0)	1.10	(0.50–2.41)	1.09	(0.49–2.42)
	3T (highest)	37	60 (30.0)	1.37	(0.63–3.00)	1.37	(0.62–3.06)
							
EBNA IgG	Negative	19	31 (15.5)	1.00	—	1.00	—
	1T (lowest)	22	57 (28.5)	0.83	(0.50–1.37)	0.78	(0.46–1.30)
	2T	31	56 (28.0)	0.94	(0.56–1.56)	0.93	(0.55–1.57)
	3T (highest)	28	56 (28.0)	0.90	(0.54–1.50)	0.87	(0.51–1.46)

aOR=adjusted odds ratio; CI=confidence interval; cOR=crude odds ratio; EA=early antigen; EBNA=EBV nuclear antigen; IgA=immunoglobulin A; IgG=immunoglobulin G;; VCA=viral capsid antigen.

a Conditional logistic regression model adjusting for age (continuous), education (none, 1–12 years, more than 13 years), alcohol consumption (never, past, current), cigarette smoking (never, past, current), and *H. pylori* antibody seropositivity (yes or no).

**Table 3:** Odds ratios of gastric adenocarcinoma risk according to the Epstein–Barr virus (EBV) antibody titres

	**Gastric adenocarcinoma (cases=87, controls=174)**			**Diagnosed ⩾2 years of enrolment (cases=63, controls=126)**		
**Antibody**	**No. cases/controls**	**aOR**	**(95% CI)^a^**	**No. cases/controls**	**aOR**	**(95% CI)^a^**
*VCA IgG*						
Negative	3/11	1.00	–	3/10	1.00	–
1T (lowest)	30/55	1.26	(0.59–2.71)	19/41	1.10	(0.48–2.49)
2T	21/52	1.15	(0.51–2.60)	15/38	1.07	(0.45–2.57)
3T (highest)	33/56	1.33	(0.59–3.00)	26/37	1.53	(0.62–3.75)
						
*EBNA IgG*						
Negative	15/29	1.00	–	11/24	1.00	–
1T (lowest)	20/48	0.87	(0.51–1.48)	14/41	0.91	(0.49–1.68)
2T	29/45	1.11	(0.64–1.92)	21/29	1.37	(0.71–2.67)
3T (highest)	23/52	0.92	(0.53–1.58)	17/32	1.23	(0.64–2.36)

aOR=adjusted odds ratio; CI=confidence interval; EBNA=EBV nuclear antigen; IgG, immunoglobulin G; VCA=viral capsid antigen.

a Conditional logistic regression model adjusting for age (continuous), education (none, 1–12 years, more than 13 years), alcohol consumption (never, past, current), cigarette smoking (never, past, current), and *H. pylori* antibody seropositivity (yes or no).
